# Klinefelter syndrome mosaicism in boys with neurodevelopmental disorders: a cohort study and an extension of the hypothesis

**DOI:** 10.1186/s13039-022-00588-z

**Published:** 2022-03-05

**Authors:** Svetlana G. Vorsanova, Irina A. Demidova, Alexey D. Kolotii, Oksana S. Kurinnaia, Victor S. Kravets, Ilya V. Soloviev, Yuri B. Yurov, Ivan Y. Iourov

**Affiliations:** 1 Veltischev Research and Clinical Institute for Pediatrics of the Pirogov Russian National Research Medical University, Ministry of Health of Russian Federation, 125412 Moscow, Russia; 2 Yurov’s Laboratory of Molecular Genetics and Cytogenomics of the Brain, Mental Health Research Center, Moscow, 115522 Russia; 3 Department of Medical Biological Disciplines, Belgorod State University, 308015 Belgorod, Russia

**Keywords:** Aneuploidy, Chromosome X, Fluorescence in situ hybridization (FISH), Klinefelter syndrome, Molecular cytogenetics, Phenotype, Somatic mosaicism

## Abstract

**Background:**

Klinefelter syndrome is a common chromosomal (aneuploidy) disorder associated with an extra X chromosome in males. Regardless of numerous studies dedicated to somatic gonosomal mosaicism, Klinefelter syndrome mosaicism (KSM) has not been systematically addressed in clinical cohorts. Here, we report on the evaluation of KSM in a large cohort of boys with neurodevelopmental disorders. Furthermore, these data have been used for an extension of the hypothesis, which we have recently proposed in a report on Turner’s syndrome mosaicism in girls with neurodevelopmental disorders.

**Results:**

Klinefelter syndrome-associated karyotypes were revealed in 49 (1.1%) of 4535 boys. Twenty one boys (0.5%) were non-mosaic 47,XXY individuals. KSM was found in 28 cases (0.6%) and manifested as mosaic aneuploidy (50,XXXXXY; 49,XXXXY; 48,XXXY; 48,XXYY; 47,XXY; and 45,X were detected in addition to 47,XXY/46,XY) and mosaic supernumerary marker chromosomes derived from chromosome X (ring chromosomes X and rearranged chromosomes X). It is noteworthy that KSM was concomitant with Rett-syndrome-like phenotypes caused by *MECP2* mutations in 5 boys (0.1%).

**Conclusion:**

Our study provides data on the occurrence of KSM in neurodevelopmental disorders among males. Accordingly, it is proposed that KSM may be a possible element of pathogenic cascades in psychiatric and neurodegenerative diseases. These observations allowed us to extend the hypothesis proposed in our previous report on the contribution of somatic gonosomal mosaicism (Turner’s syndrome mosaicism) to the etiology of neurodevelopmental disorders. Thus, it seems to be important to monitor KSM (a possible risk factor or a biomarker for adult-onset multifactorial brain diseases) and analysis of neuromarkers for aging in individuals with Klinefelter syndrome. Cases of two or more supernumerary chromosomes X were all associated with KSM. Finally, Rett syndrome-like phenotypes associated with KSM appear to be more common in males with neurodevelopmental disorders than previously recognized.

## Background

In 1942, H.F. Klinefelter, E.C. Reifenstein and F. Albright described a clinical condition, which is now known as Klinefelter syndrome [1]. In 1959, P.A. Jacobs and J.A. Strong proposed an “XXY sex-determining mechanism” for the condition and suggested the presence of an extra X chromosome to be the cause for the syndrome [2]. Nowadays, Klinefelter syndrome is considered the commonest aneuploidy syndrome in males affecting 1:500–1:700 male newborns [3–5]. Karyotypic heterogeneity (i.e. chromosomal abnormalities producing the effect of additional chromosome(s) X) is vast in Klinefelter syndrome. In addition to 47,XXY in the majority of cases, Klinefelter syndrome-associated chromosomal abnormalities may be non-mosaic or mosaic aneuploidy (48,XXXY, 48,XXYY, 48,XYYY, 49,XXXXY, 49,XXXYY, 49XYYYY, 50,XXXXXY etc.) and supernumerary rearranged chromosomes X (supernumerary marker chromosomes derived from chromosome X) [5, 6]. Still, there is an opinion that several additional gonosomes in males (non-mosaic and mosaic) and Klinefelter syndrome mosaicism (KSM) may cause sex chromosome aneuploidy syndromes or conditions other than Klinefelter syndrome [5]. Moreover, somatic gonosomal mosaicism appears to be involved in the pathogenesis of complex diseases (e.g. complex brain disorders) [7, 8]. Alternatively, the effect of an extra X chromosome is likely to be associated with multifactorial brain diseases (autism and schizophrenia) in Klinefelter syndrome individuals [9]. Additionally, 47,XXY/46,XY mosaicism is common in autistic children, whereas non-mosaic 47,XXY karyotype is detectable in children and adolescents with Klinefelter syndrome and autism [10–12]. Schizophrenia is significantly more common in Klinefelter syndrome individuals than in the general population [13]. 47,XXY karyotype or Klinefelter syndrome is suggested to be an important risk factor for psychosis, autism and attention-deficit hyperactivity disorder [14]. Usually, the contribution of an extra X chromosome to brain diseases is addressed by studying individuals with Klinefelter syndrome [11, 13, 14]. The inverse study design (i.e. analysis of 47,XXY karyotype in a clinical cohort) is much more rare [10, 15, 16]. Moreover, similar studies of KSM are even rarer [7, 10]. Previously, KSM have been shown to be almost the commonest genetic alternation in children with idiopathic autism [10, 12]. In total, KSM may be an important contributor to the etiology of neurodevelopmental disorders, as a whole.

In the present report, we describe the study of KSM in a large cohort of boys with neurodevelopmental disorders and congenital anomalies by molecular cytogenetic techniques. Karyotypic, molecular and clinical data have been used for evaluating possible phenotypic outcomes of KSM. Furthermore, using these data, we found possible to extend our recent hypothesis concerning diagnostic and prognostic significance of ontogenetic instability of the X chromosome (for more details, see [8]).

## Materials and methods

### Patients

The cohort of boys with neurodevelopmental disorders (intellectual disability, autism and/or epilepsy) and congenital anomalies included 4535 individuals. The ages ranged between 1 month and 18 years (mean age: 8.4 years). Molecular (cyto)genetic studies of the cohort were approved by the Ethics Committee of the Veltischev Research and Clinical Institute for Pediatrics of the Pirogov Russian National Research Medical University, Ministry of Health of Russian Federation, Moscow. Written informed consent was obtained from the parents of the patients.

### Cytogenetic analysis

Karyotyping by G- and C-banding was performed for all the boys from the cohort as described previously [17–19]. The resolution of G-banding was no less than 550 bands (for details, see ISCN 2020 [20]).

### FISH

KSM was evaluated by fluorescence in situ hybridization (FISH) with chromosome-enumeration and site-specific DNA probes. X-chromosome-specific DNA probe DXZ1 was used in all the cases, which demonstrated additional chromosome X (mosaic and non-mosaic cases). X chromosome site-specific DNA probes (suggested structural X chromosome rearrangements) and chromosome-enumeration DNA probes for autosomes and chromosome Y (marker chromosomes and controlling in KSM analysis) were applied when needed. Protocols for DNA probe labeling and FISH (hybridization and detection) including interphase FISH analysis was previously described in details [21–24]. Quantitative FISH was used for interphase analyses for increasing the efficiency of aneuploidy scoring [25, 26]. FISH analysis was performed in 513 patients out of whole cohort, which encompassed all cases of KSM. No fewer than 100 metaphase spreads and 1200 interphase nuclei were scored per case.

### SNP-array

Molecular karyotyping by SNP-array was performed using CytoScan HD Arrays (Affymetrix, Santa Clara, CA, USA; ~ 2.7 million markers) as described earlier [27, 28]. Affymetrix ChAS (Chromosome Analysis Suite) software (CytoScan® HD Array Version 4.1.0.90/r29400; reference sequence—GRCh37/hg19) was applied for data visualization. Out of the whole cohort, 372 patients with severe intellectual disability and multiple congenital malformations were analyzed.

## Results

**Fig. 1:**
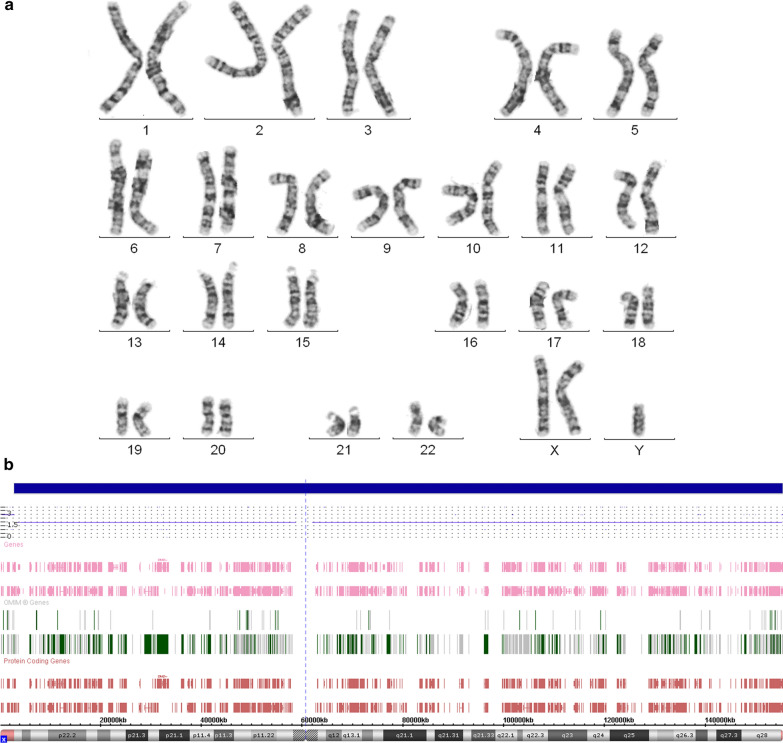
Cytogenetic and cytogenomic findings in a non-mosaic case of 47,XXY; **a** G-banding of metaphase chromosomes; **b** SNP-array results demonstrating additional chromosome X in male

Klinefelter syndrome-associated karyotypes were observed in 49 (1.1%) out of 4535 boys with neurodevelopmental disorders and congenital anomalies. Non-mosaic 47,XXY karyotypes (Fig. 1) were identified in 21 boys (0.5% or 42.8% out of the whole group or boys with Klienfelter syndrome-associated karyotypes, respectively). Among these cases, one was 48,XXY, + 21 (i.e. c-occurrence of Klienfelter and Down syndromes). KSM was uncovered in 28 boys (0.6% or 57.2% out of the whole group or boys with Klinefelter syndrome-associated karyotypes, respectively); cells with 47,XXY karyotypes were detected in all these cases. Mosaicism rates varied from 1 to 95%. SNP array analysis did not demonstrate copy number variants which might be associated with neurodevelopmental disorders in patients with Klienfelter syndrome-associated karyotypes including KSM cases. Four cases demonstrated non-mosaic 47,XXY, whereas other cases were featured by molecular karyotypes, which were not associated with neurodevelopmental disorders.

**Fig. 2:**
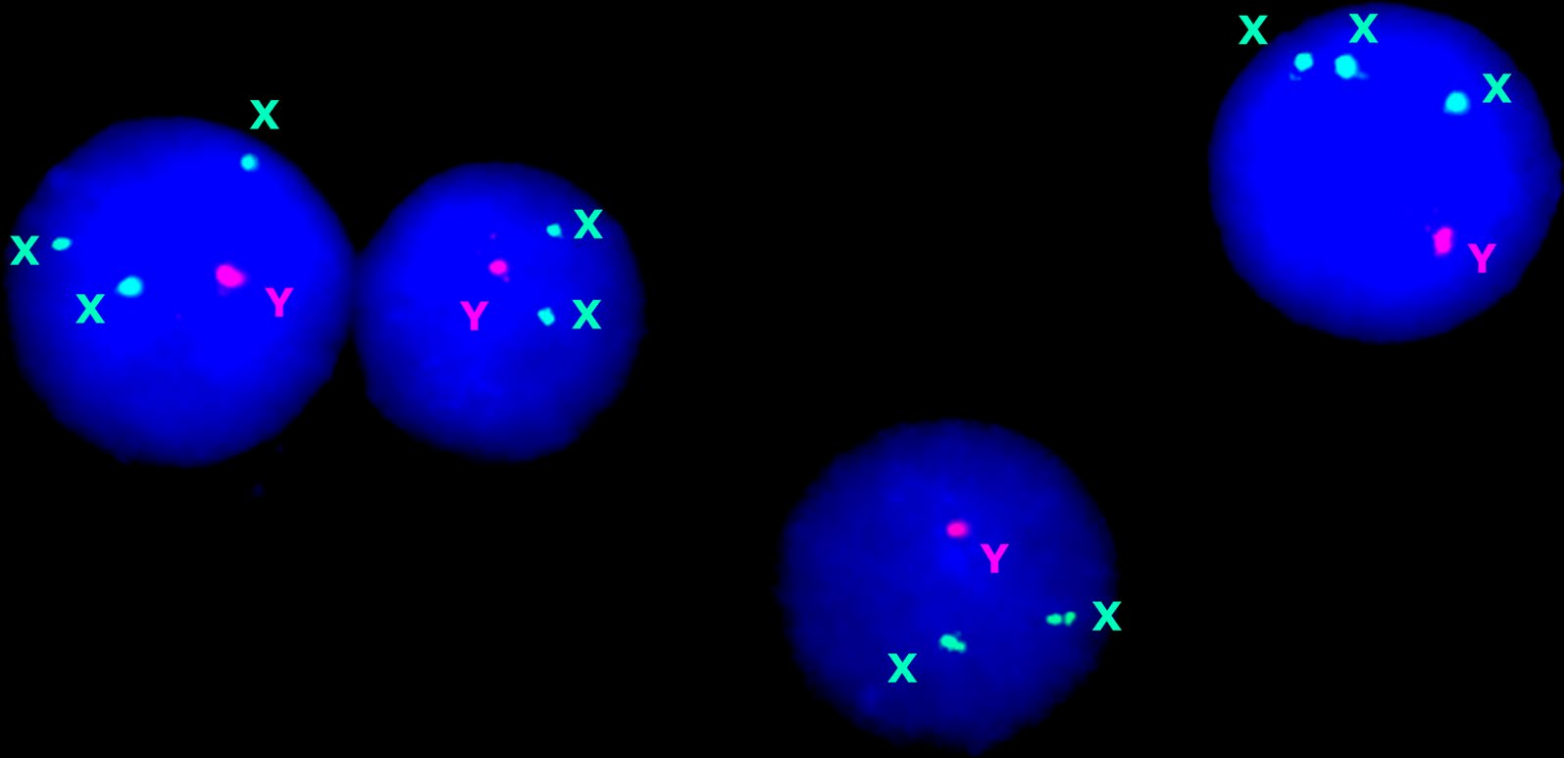
Interphase FISH with chromosome-enumeration probes for chromosomes X (DXZ1) and Y (DYZ3)

**Fig. 3:**
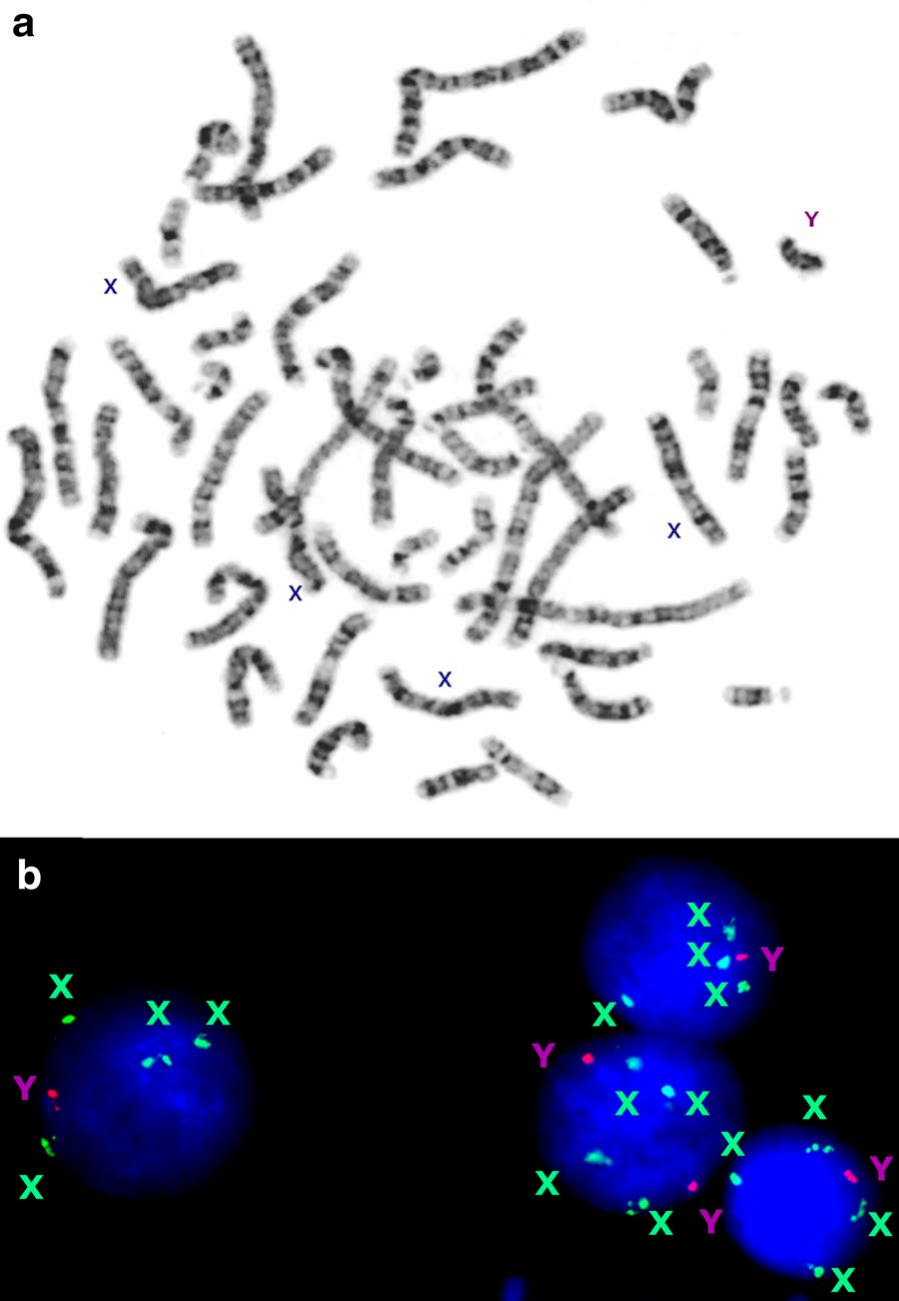
Cytogenetic and molecular cytogenetic findings in a case of 49,XXXXY; **a** G-banded metaphase spread demonstrating three additional chromosomes X in a male; **b** Interphase FISH with chromosome-enumeration probes for chromosomes X (DXZ1) and Y (DYZ3) demonstrating mosaic aneuploidies

**Fig. 4:**
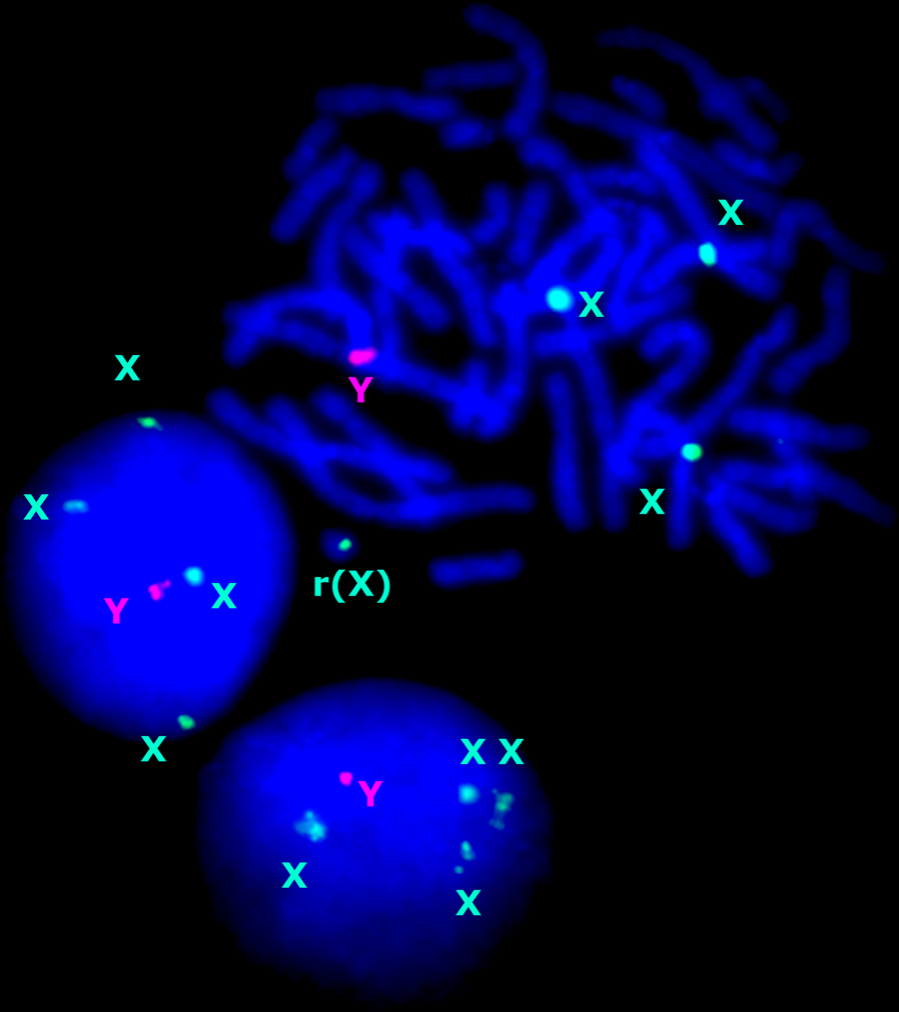
FISH with chromosome-enumeration probes for chromosomes X (DXZ1) and Y (DYZ3) demonstrating gonosomal aneuploidy and a ring chromosome X

All the KSM cases were confirmed by interphase FISH (Fig. 2). In addition to cells with 47,XXY karyotypes, we detected cells with following aneuploidies: 50,XXXXXY, 49,XXXXY (Fig. 3), 48,XXXY, 48,XXYY, 47,XXY, and 45,X. We also detected Klinefelter syndrome-associated karyotypes featured by structural rearrangements of additional chromosomes X: 47,XYr(X) and 47,XY, + mar, where mar = der(X). A case of supernumerary ring chromosome X was mosaic (47,XYr(X)—7%, 49,XXXYr(X)—11% (Fig. 4); 47,XXY—30%, 46,XY—52%). Supernumerary maker chromosomes were found in two cases. One case was mosaic (supernumerary rearranged chromosome X—10.5%; 45,X—2.6%; 47,XYY—3.7%; 46,XY—83.2%), whereas another case was non-mosaic. Overview of KSM cases is provided in Table 1.

**Table 1:** Overview of KSM cases

Chromosome complements	*Cell proportions (%)	Brief phenotypical overview
47,XXY/46,XY	9/91	Mild intellectual disability, disorder of sex development
47,XXY/46,XY	95/5	Severe intellectual disability, autism, congenital malformations, Klinefelter syndrome features
47,XXY/46,XY	12/88	Rett-syndrome-like phenotype
47,XXY/46,XY	7/93	Fragile X syndrome, autism
48,XXXY/50,XXXXXY/ 49,XXXXY/47,XXY	50/20/18/12	Severe intellectual disability, disorder of sex development, multiple congenital malformations
47,XXY/46,XY	4/96	Mild intellectual disability, Klinefelter syndrome features
49,XXXXY/46,XXY	91/9	Fragile X syndrome, severe intellectual disability, disorder of sex development, multiple congenital malformations
47,XXY/46XY	7/93	Rett-syndrome-like phenotype
49,XXXXY/48,XXXY/ 47,XXY	74//19/7	Severe intellectual disability, multiple congenital malformations, skeletal dysplasia
47,XXY/46,XY	91/9	Mild intellectual disability, Klinefelter syndrome features
47,XXY/46,XY	9/91	Rett-syndrome-like phenotype
47,XXY/46,XY	7/93	Rett-syndrome-like phenotype
47,XXY/46,XY	7/93	Intellectual disability, congenital malformations
47,XXY/45,X/47,XYY/46,XY	8/5/4/83	Intellectual disability, microcephaly, multiple congenital malformations
47,XXY/45,X/46,XY	13/8/79	Intellectual disability, autism, multiple congenital malformations
47,XXY/45,X/46XY	8/7/85	Intellectual disability, epilepsy, congenital heart defect (Williams-Beuren syndrome)
47,XXY/48,XXXY/46,XY	50/37/13	Intellectual disability, Klinefelter syndrome features, myopia
48,XXYY/47,XXY/46,XY	62/15/23	Intellectual disability, disorder of sex development
47,XXY/45,X/46,XY	21/18/61	Mild intellectual disability, disorder of sex development
47,XY,r(X)/46,XY	16/84	Mild intellectual disability, disorder of sex development
47,XXY/46,XY	99/1	Mild intellectual disability, Klinefelter syndrome features
49,XXXXY/47,XXY/48,XXXY	87/7/6	Severe intellectual disability, multiple congenital malformations, disorder of sex development
49,XXXXY/50,XXXXXУ/ 47,XXY	91/6/3	Intellectual disability, muscular hypotonia, obesity, disorder of sex development
48,XXYY/47,XXУ/47,XYY	95/3/2	Intellectual disability, autism, aggressive behavior, multiple congenital malformations
47,XXY/47,XYY/46XY	5/1/94	Gilbert syndrome, Mild intellectual disability, congenital malformations
47,XXY/46,XY	8/92	CDKL5-deficiency (severe intellectual disability, epilepsy)
49,XXXXY/47,XXY	91/9	Severe intellectual disability, multiple congenital malformations, congenital heart defect, disorder of sex development
47,XXY/46,XY	30/70	Rett-syndrome-like phenotype

^*^According to FISH analysis

Among cases of KSM, ~ 50% were those expressing low-level mosaicism (< 20% of cells affected by aneuploidy). The total amount of cells (metaphase plates + interphase nuclei) scored for all the individuals exceeded 25,000. The distribution of non-aneuploid and aneuoloid cells was as follows: cells without gonosomal aneuploidy—50.4% and aneuploid cells—49.6%. The distribution among aneuploid cells was determined as follows: XXY—21.2%, XXXXY—8.1%, XXYY—6.6%, + der(x)—4.4%, XXXY—4.4%, one chromosome X—2%, XXXXXY—1.2%, XYY—1%, ring chromosome X—0.7%.

Phenotypically, individuals affected by non-mosaic 47,XXY exhibited Klinefelter syndrome with a variety of neurobehavioral abnormalities (e.g. mild intellectual disability and autism). Among individuals with Klinefelter syndrome-associated karyotypes, 81.6% demonstrated abnormalities in sexual development. Clinically, individuals with KSM demonstrated a wide spectrum of conditions ranged from severe to mild intellectual disability and congenital anomalies. KSM was frequently concomitant with other genetic diseases. Thus, Rett-syndrome-like phenotypes (i.e. Rett syndrome in males) caused by *MECP2* mutations concomitant with KSM were observed in 5 cases (0.1%); two cases exhibited fragile X syndrome (*FMR1* repeat expansions were molecularly confirmed) with KSM; a case of *CDKL5* mutation (C532C > T) causing CDKL5-deficiency demonstrated KSM; single cases of KSM were concomitant with Gilbert and Williams-Beuren syndromes (cytogenetically/molecularly confirmed). No phenotype-karyotype correlations (correlations between clinical outcomes and KSM levels) were found (for more details, see Table 1). The age of individuals with Klinefelter syndrome-associated karyotypes was generally lower than 6 years. Consequently, significant correlations between changes in KSM levels and age were impossible to obtain.

## Discussion

Klinefelter syndrome (47,XXY) is the commonest gonosomal syndrome in males [29]. Additional chromosome X in males possesses an appreciable impact on brain functioning, the manifestations of which range from mild cognitive difficulties to severe neuropsychiatric disorders [30–33]. Unfortunately, similar effects have not been systematically evaluated in cases of KSM [5, 34]. However, taking into account the effect of gonosomal aneuploidy (non-mosaic and mosaic) on brain functioning, in general [16, 35], a role of KSM in brain dysfunction is expected. Moreover, dynamic nature of somatic chromosomal mosaicism leads to the involvement in pathogenetic and ontogenetic processes [7, 36, 37]. These processes are further involved in intercellular genetic (genomic) diversity [35, 38, 39], early-onset brain diseases [7, 10, 28, 40, 41], late-onset brain diseases [34, 42–46], behavior [47], and aging [43, 48–51]. Therefore, the analysis of KSM in the context of brain diseases seems to be required. Accordingly, we took an opportunity to evaluate KSM in a large neurodevelopmental cohort started to be described decades ago [10, 17, 52]. Thus, rates of Klinefelter syndrome-associated karyotypes and KSM among boys with neurodevelopmental disorders were determined as 1.1% and 0.6%, respectively, for the first time. Taking into account the occurrence of neurodevelopmental disorders, one may conclude that KSM is likely to be involved in the pathogenesis. Karyotypic variations among individuals with KSM allowed us to conclude that this type of somatic chromosomal mosaicism has a highly dynamic nature. The latter has been recently proposed to be an important factor for evaluating mechanisms and possible therapies of a disease [37]. Additionally, dynamic nature of KSM underlies the lack of phenotype-karyotype correlations (correlations between mosaicism rates and phenotypic outcomes).

Another intriguing observation is the frequent concomitance of KSM with other genetic diseases. Rett-syndrome-like phenotypes caused by *MECP2* mutations co-occurred rather frequently with KSM (0.1%) among boys with neurodevelopmental disorders. Although Klinefelter and Rett syndromes are relatively frequent genetic diseases, the uncovered frequency of the co-occurrence is not likely to be a coincidence. It is generally accepted that additional chromosome X is required for the survival of males affected by X-linked dominant mutations/disorders (e.g. Rett syndrome and CDKL5 deficiency). This fact is the most probable explanation for the frequent occurrence of Rett-syndrome-like phenotypes demonstrating KSM [17, 53, 54]. Nonetheless, it should be concluded that Rett-syndrome-like phenotypes caused by *MECP2* mutations and KSM are more frequent among males with neurodevelopmental disorders than previously recognized. A case was associated with CDKL5-deficiency (*CDKL5* mutation; C532C > T) and KSM. Since CDKL5-deficiency may be considered as an atypical Rett-syndrome-like disorder [55], Rett-syndrome-like phenotypes associated with KSM may be even more frequent among males with neurodevelopmental disorders. Cases that demonstrated co-occurrence of *CDKL5* mutations and Klinefelter syndrome-associated karyotypes were previously reported [56]. *FMR1* repeat expansions co-occurred with KSM in two cases. Similar concomitance was previously reported [57, 58]. Non-mosaic Klinefelter and Down syndromes co-occur rather frequently inasmuch as these are the commonest chromosomal disorders [18]. Certainly, the phenotypes in these cases are essentially the result of X-linked mutations and trisomy of chromosome 21. However, the phenotypic effect of an additional chromosome X in a male karyotype may not be excluded.

Since no direct phenotype-karyotype correlations were revealed, we suggested KSM to be rather an element of the pathogenetic cascade than a phenotype-causing genetic change per se. The idea is supported by the reports demonstrating high frequency of mosaic 47,XXY in children with neurodevelopmental (neurobehavioral) disorders [10, 12, 17, 19, 47]. Supernumerary chromosome X seems to be a critical element of the pathogenetic cascade of psychiatric diseases observed with high frequency in Klinefelter syndrome [5, 11, 13–16]. Moreover, since aneuploidy levels are age-dependent and are involved in normal and pathogenic aging [7, 37, 48–51], it is highly likely that KSM levels are able to change with age. The dynamic nature of KSM exhibited by cases associated with multiple aneuploidy and structurally rearranged chromosomes X supports this assumption. Unfortunately, neither age-dependent KSM variation nor brain aging in individuals with Klinefelter syndrome-associated karyotypes has been addressed.

The gap in our knowledge concerning possible involvement of KSM in pathogenesis of brain diseases is likely to result from the lack of technological solutions. Molecular cytogenetic techniques provide for high-resolution single-cell analysis of somatic chromosomal mosaicism at all the stages of cell cycle (i.e. in any tissue) [7, 39]. However, since there is a need to understand molecular pathways leading to and affected by somatic chromosomal mosaicism and chromosome instability [59], it is strongly recommended to process molecular cytogenetic and cytogenomic data by bioinformatic (system biology) technologies [60, 61]. These data may allow to unravel molecular and cellular pathways to KSM as well as decipher KSM impact on cellular/tissular physiology [39, 61]. This knowledge is likely to become significant for developing techniques of molecular diagnosis of brain diseases mediated by somatic chromosomal mosaicism and chromosome instability, which has been previously shown important for biomedical and diagnostic research [62]. Our findings and discussions concerning KSM in boys with neurodevelopmental disorders correlated with a hypothesis concerning the role of ontogenetic changes in the levels of gonosomal aneuploidy (Turner’s syndrome mosaicism) in brain dysfunction [8]. Consequently, we found possible to extend the hypothesis further.

## Hypothesis extended

Recently, we have proposed a hypothesis suggesting ontogenetic changes in the levels of gonosomal aneuploidy to be a possible mechanism for complex brain diseases. More precisely, X chromosome loss was suggested to increase throughout the lifespan leading, thereby, to occurrence of complex diseases associated with aneuploidy in later life [8]. Similarly, mosaic X chromosome gain in males or KSM is likely associated with ontogenetic variations in the rates of mosaicism. As mentioned previously, the increase, which may be mediated by alterations to genome safeguarding pathways and genetic-environmental interactions, has to lead to manifestations of diseases associated with X aneuploidy (gain) in males. We hypothesize these diseases to be neurobehavioral disorders (especially, autism and intellectual disability), schizophrenia and, probably, dementia (e.g. Alzheimer’s disease). Since these brain disorders are of increasing socio-medical significance, KSM analysis might be an important part of early (preclinical) diagnosis, prognosis and possible therapeutic interventions. As previously suggested for Turner’s syndrome mosaicism [8], molecular cytogenetic monitoring of KSM for early detection of changes in the X chromosome aneuploidy rates is recommended. Moreover, bioinformatic analyses (system biology studies) of molecular and cellular pathways leading to the X chromosome aneuploidy/instability might provide an opportunity for controlling (inhibiting) somatic chromosomal mosaicism and/or chromosome instability. Furthermore, systems biology studies of consequences of KSM (Klinefelter syndrome-associated karyotypes) might shed light on the effect of additional chromosomes X in males suffering from psychiatric and neurodegenerative diseases. These studies might gain more relevance by combination of molecular cytogenetic and bioinformatic assessments of ontogenetic changes in KSM levels. Molecular cytogenetic monitoring and systems biology analysis of causes and consequences of KSM is able to provide a successful evidence-based therapy of devastating multifactorial brain diseases.

## Conclusions

Klinefelter syndrome-associated karyotypes affect 1.1% of boys with neurodevelopmental disorders (~ 10 in 1000 boys suffering from intellectual disability, autism, epilepsy and/or congenital anomalies). Cases of two or more supernumerary chromosomes X (i.e. XXXY, XXXXY, XXXXXY etc.) were all associated with KSM. X-linked dominant mutations in *MECP2* (Rett syndrome) and *CDKL5* (CDKL5 deficiency) genes are frequently associated with KSM in males with neurodevelopmental disorders. More importantly, Rett-syndrome-like phenotypes concomitant with KSM seem to be more common than previously recognized (0.1%). Significant heterogeneity in chromosomal complements mediated by KSM was observed in males with neurodevelopmental disorders. Accordingly, KSM may be recognized as a contributor to the risk of brain disorders. It appears that studies dedicated to ontogenetic changes in KSM levels and to effects of Klinefelter syndrome-associated karyotypes in late ontogeny are required. Still, our observations on KSM in boys with neurodevelopmental disorders allowed us to extend our hypothesis proposed previously for Turner’s syndrome mosaicism [8]. KSM proportions are likely to change through the ontogeny in favor of the abnormal cells. Therefore, KSM might be a biomarker for adult-onset (multifactorial) brain diseases, which are mediated by X chromosome mosaicism. Thus, the detection and monitoring of gonosomal mosaicism is important for early diagnosis, prognosis and evidence-based therapeutic interventions in brain diseases.

## Data Availability

The data of this study are all included in the article.

## References

[1] Klinefelter HF, Reifenstein EC, Albright F (1942). Syndrome characterized by gynecomastia, aspermatogenesis without a leydigism and increased secretion of follicle-stimulating hormone. J Clin Endocrinol Metab.

[2] Jacobs PA, Strong JA (1959). A case of human intersexuality having a possible XXY sex-determining mechanism. Nature.

[3] Lanfranco F, Kamischke A, Zitzmann M, Nieschlag E (2004). Klinefelter's syndrome. Lancet.

[4] Groth KA, Skakkebæk A, Høst C, Gravholt CH, Bojesen A (2013). Clinical review: Klinefelter syndrome—a clinical update. J Clin Endocrinol Metab.

[5] Garolla A, Corona G. (eds) Klinefelter’s syndrome—from a disabling condition to a variant of normalcy. Trends in Andrology and Sexual Medicine. Springer, Cham, 2020.

[6] Fruhmesser A, Kotzot D (2011). Chromosomal variants in Klinefelter syndrome. Sex Dev.

[7] Iourov IY, Vorsanova SG, Yurov YB, Kutsev SI (2019). Ontogenetic and pathogenetic views on somatic chromosomal mosaicism. Genes (Basel).

[8] Vorsanova SG, Kolotii AD, Kurinnaia OS, Kravets VS, Demidova IA, Soloviev IV, Yurov YB, Iourov IY (2021). Turner's syndrome mosaicism in girls with neurodevelopmental disorders: a cohort study and hypothesis. Mol Cytogenet.

[9] Skakkebaek A, Viuff M, Nielsen MM, Gravholt CH (2020). Epigenetics and genomics in Klinefelter syndrome. Am J Med Genet C Semin Med Genet.

[10] Yurov YB, Vorsanova SG, Iourov IY, Demidova IA, Beresheva AK, Kravetz VS, Monakhov VV, Kolotii AD, Voinova-Ulas VY, Gorbachevskaya NL (2007). Unexplained autism is frequently associated with low-level mosaic aneuploidy. J Med Genet.

[11] Jha P, Sheth D, Ghaziuddin M (2007). Autism spectrum disorder and Klinefelter syndrome. Eur Child Adolesc Psychiatry.

[12] Vorsanova SG, Yurov IY, Demidova IA, Voinova-Ulas VY, Kravets VS, Solov'ev IV, Gorbachevskaya NL, Yurov YB (2007). Variability in the heterochromatin regions of the chromosomes and chromosomal anomalies in children with autism: identification of genetic markers of autistic spectrum disorders. Neurosci Behav Physiol.

[13] DeLisi LE, Maurizio AM, Svetina C, Ardekani B, Szulc K, Nierenberg J, Leonard J, Harvey PD (2005). Klinefelter’s syndrome (XXY) as a genetic model for psychotic disorders. Am J Med Genet B Neuropsychiatr Genet.

[14] Cederlöf M, Ohlsson Gotby A, Larsson H, Serlachius E, Boman M, Långström N, Landén M, Lichtenstein P (2014). Klinefelter syndrome and risk of psychosis, autism and ADHD. J Psychiatr Res.

[15] Demirhan O, Taştemir D (2003). Chromosome aberrations in a schizophrenia population. Schizophr Res.

[16] Graham EJ, Vermeulen M, Vardarajan B, Bennett D, de Jager P, Pearse RV 2nd, Young-Pearse TL, Mostafavi S. Somatic mosaicism of sex chromosomes in the blood and brain. Brain Res. 2019;1721:146345. 10.1016/j.brainres.2019.146345 PMC6717667 31348909

[17] Vorsanova SG, Yurov YB, Ulas VY, Demidova IA, Sharonin VO, Kolotii AD, Gorbatchevskaia NL, Beresheva AK, Soloviev IV (2001). Cytogenetic and molecular-cytogenetic studies of Rett syndrome (RTT): a retrospective analysis of a Russian cohort of RTT patients (the investigation of 57 girls and three boys). Brain Dev.

[18] Vorsanova SG, Iourov IY, Beresheva AK, Demidova IA, Monakhov VV, Kravets VS, Bartseva OB, Goyko EA, Soloviev IV, Yurov YB (2005). Non-disjunction of chromosome 21, alphoid DNA variation, and sociogenetic features of Down syndrome. Tsitol Genet.

[19] Vorsanova SG, Voinova VY, Yurov IY, Kurinnaya OS, Demidova IA, Yurov YB (2010). Cytogenetic, molecular-cytogenetic, and clinical-genealogical studies of the mothers of children with autism: a search for familial genetic markers for autistic disorders. Neurosci Behav Physiol.

[20] McGowan-Jordan J, Hastings RJ, Moore S, eds. ISCN 2020—An International System for Human Cytogenomic Nomenclature (2020). Karger, 2020.

[21] Yurov YB, Vostrikov VM, Vorsanova SG, Monakhov VV, Iourov IY (2001). Multicolor fluorescent in situ hybridization on post-mortem brain in schizophrenia as an approach for identification of low-level chromosomal aneuploidy in neuropsychiatric diseases. Brain Dev.

[22] Iourov IY, Liehr T, Vorsanova SG, Kolotii AD, Yurov YB (2006). Visualization of interphase chromosomes in postmitotic cells of the human brain by multicolour banding (MCB). Chromosome Res.

[23] Yurov YB, Vorsanova SG, Soloviev IV, Ratnikov AM, Iourov IY (2017). FISH-based assays for detecting genomic (chromosomal) mosaicism in human brain cells. NeuroMethods.

[24] Yurov YB, Vorsanova SG, Demidova IA, Kolotii AD, Soloviev IV, Iourov IY (2018). Mosaic brain aneuploidy in mental illnesses: an association of low-level post- zygotic aneuploidy with schizophrenia and comorbid psychiatric disorders. Curr Genomics.

[25] Iourov IY, Soloviev IV, Vorsanova SG, Monakhov VV, Yurov YB (2005). An approach for quantitative assessment of fluorescence in situ hybridization (FISH) signals for applied human molecular cytogenetics. J Histochem Cytochem.

[26] Iourov IY (2017). Quantitative fluorescence in situ hybridization (QFISH). Methods Mol Biol.

[27] Iourov IY, Vorsanova SG, Korostelev SA, Zelenova MA, Yurov YB (2015). Long contiguous stretches of homozygosity spanning shortly the imprinted loci are associated with intellectual disability, autism and/or epilepsy. Mol Cytogenet.

[28] Iourov IY, Vorsanova SG, Yurov YB, Zelenova MA, Kurinnaia OS, Vasin KS, Kutsev SI (2020). The cytogenomic "theory of everything": chromohelkosis may underlie chromosomal instability and mosaicism in disease and aging. Int J Mol Sci.

[29] Berglund A, Stochholm K, Gravholt CH (2020). The epidemiology of sex chromosome abnormalities. Am J Med Genet C Semin Med Genet.

[30] Savic I (2012). Advances in research on the neurological and neuropsychiatric phenotype of Klinefelter syndrome. Curr Opin Neurol.

[31] Skakkebæk A, Moore PJ, Pedersen AD, Bojesen A, Kristensen MK, Fedder J, Laurberg P, Hertz JM, Østergaard JR, Wallentin M, Gravholt CH (2017). The role of genes, intelligence, personality, and social engagement in cognitive performance in Klinefelter syndrome. Brain Behav.

[32] van Rijn S, de Sonneville L, Swaab H (2018). The nature of social cognitive deficits in children and adults with Klinefelter syndrome (47, XXY). Genes Brain Behav.

[33] Whitman ET, Liu S, Torres E, Warling A, Wilson K, Nadig A, McDermott C, Clasen LS, Blumenthal JD, Lalonde FM, Gotts SJ, Martin A, Raznahan A (2021). Resting-state functional connectivity and psychopathology in Klinefelter syndrome (47, XXY). Cereb Cortex.

[34] Iourov IY, Vorsanova SG, Yurov YB (2006). Chromosomal variation in mammalian neuronal cells: known facts and attractive hypotheses. Int Rev Cytol.

[35] Printzlau F, Wolstencroft J, Skuse DH (2017). Cognitive, behavioral, and neural consequences of sex chromosome aneuploidy. J Neurosci Res.

[36] Iourov IY, Vorsanova SG, Yurov YB (2013). Somatic cell genomics of brain disorders: a new opportunity to clarify genetic-environmental interactions. Cytogenet Genome Res.

[37] Vorsanova SG, Yurov YB, Iourov IY (2020). Dynamic nature of somatic chromosomal mosaicism, genetic-environmental interactions and therapeutic opportunities in disease and aging. Mol Cytogenet.

[38] Hultén MA, Jonasson J, Iwarsson E, Uppal P, Vorsanova SG, Yurov YB, Iourov IY (2013). Trisomy 21 mosaicism: we may all have a touch of Down syndrome. Cytogenet Genome Res.

[39] Iourov IY, Vorsanova SG, Yurov YB (2012). Single cell genomics of the brain: focus on neuronal diversity and neuropsychiatric diseases. Curr Genomics.

[40] Potter H, Chial HJ, Caneus J, Elos M, Elder N, Borysov S, Granic A (2019). Chromosome instability and mosaic aneuploidy in neurodegenerative and neurodevelopmental disorders. Front Genet.

[41] Iourov IY, Vorsanova SG, Kurinnaia OS, Zelenova MA, Vasin KS, Yurov YB (2021). Causes and consequences of genome instability in psychiatric and neurodegenerative diseases. Mol Biol.

[42] Yurov YB, Vorsanova SG, Iourov IY (2011). The DNA replication stress hypothesis of Alzheimer's disease. ScientificWorldJournal.

[43] Kennedy SR, Loeb LA, Herr AJ (2012). Somatic mutations in aging, cancer and neurodegeneration. Mech Ageing Dev.

[44] Yurov YB, Vorsanova SG, Liehr T, Kolotii AD, Iourov IY (2014). X chromosome aneuploidy in the Alzheimer's disease brain. Mol Cytogenet.

[45] Yurov YB, Vorsanova SG, Iourov IY (2019). Chromosome instability in the neurodegenerating brain. Front Genet.

[46] Bajic VP, Essack M, Zivkovic L, Stewart A, Zafirovic S, Bajic VB, Gojobori T, Isenovic E, Spremo-Potparevic B (2020). The X Files: "The mystery of X chromosome instability in Alzheimer's disease". Front Genet.

[47] Vorsanova SG, Zelenova MA, Yurov YB, Iourov IY (2018). Behavioral variability and somatic mosaicism: A cytogenomic hypothesis. Curr Genomics.

[48] Yurov YB, Vorsanova SG, Iourov IY (2009). GIN'n'CIN hypothesis of brain aging: deciphering the role of somatic genetic instabilities and neural aneuploidy during ontogeny. Mol Cytogenet.

[49] Yurov YB, Vorsanova SG, Iourov IY (2010). Ontogenetic variation of the human genome. Curr Genomics.

[50] Vijg J (2014). Somatic mutations, genome mosaicism, cancer and aging. Curr Opin Genet Dev.

[51] Iourov IY, Yurov YB, Vorsanova SG, Kutsev SI (2021). Chromosome instability, aging and brain diseases. Cells.

[52] Vorsanova SG, Iurov IIu, Kurinnaia OS, Voinova VIu, Iurov IuB (2013). Genomic abnormalities in children with mental retardation and autism: the use of comparative genomic hybridization in situ (HRCGH) and molecular karyotyping with DNA-microchips (array CGH). Zh Nevrol Psikhiatr Im S S Korsakova.

[53] Vorsanova SG, Iourov IY, Yurov YB (2004). Neurological, genetic and epigenetic features of Rett syndrome. J Pediatr Neurol.

[54] Iourov IY, Vorsanova SG, Voinova VY, Kurinnaia OS, Zelenova MA, Demidova IA, Yurov YB (2013). Xq28 (MECP2) microdeletions are common in mutation-negative females with Rett syndrome and cause mild subtypes of the disease. Mol Cytogenet.

[55] Fehr S, Wilson M, Downs J, Williams S, Murgia A, Sartori S, Vecchi M, Ho G, Polli R, Psoni S, Bao X, de Klerk N, Leonard H, Christodoulou J (2013). The CDKL5 disorder is an independent clinical entity associated with early-onset encephalopathy. Eur J Hum Genet.

[56] Sartori S, Di Rosa G, Polli R, Bettella E, Tricomi G, Tortorella G, Murgia A (2009). A novel CDKL5 mutation in a 47, XXY boy with the early-onset seizure variant of Rett syndrome. Am J Med Genet A.

[57] Bodurtha J, Jackson-Cook C, Maddalena A, Piserchio J, Waller R (1993). 46XY/47XYY mosaicism and fragile X. Clin Genet.

[58] Banes SL, Begleiter ML, Butler MG (2003). 45, X/46, XY mosaicism and fragile X syndrome. Am J Med Genet A.

[59] Iourov IY, Vorsanova SG, Yurov YB (2019). Pathway-based classification of genetic diseases. Mol Cytogenet.

[60] Iourov IY (2019). Cytopostgenomics: What is it and how does it work?. Curr Genomics.

[61] Iourov IY, Vorsanova SG, Yurov YB (2019). The variome concept: focus on CNVariome. Mol Cytogenet.

[62] Vorsanova SG, Yurov YB, Soloviev IV, Iourov IY (2010). Molecular cytogenetic diagnosis and somatic genome variations. Curr Genomics.

